# Supplementary material for: The genetic interaction of REVOLUTA and WRKY53 links plant development, senescence, and immune responses
Source: PLoS One. 2022 Mar 25;17(3):e0254741. doi: 10.1371/journal.pone.0254741 (PMC8956159; doi:10.1371/journal.pone.0254741)
Supplement: S1 Table — Percentages indicate the percentage the total variance explained in the three first principal components (PC). Loadings are correlation coefficients between the variables and PCs. DM = dry mass. (DOCX) [file pone.0254741.s001.docx]

**S1 Table. Loadings of the variables included in the PCA on mean of metabolite abundance values.** Percentages indicate the percentage the total variance explained in the three first principal components (PC). Loadings are correlation coefficients between the variables and PCs. DM = dry mass

| **Trait (unit)** | **PC1** | **PC2** | **PC3** |
| --- | --- | --- | --- |
|  | **30.4%** | **20.1%** | **17.3%** |
| Camalexin (ng mg^-1^ DM) | 0.87 | -0.14 | -0.23 |
| Salicyl acid (SA; ng mg^-1^ DM) | 0.86 | 0.062 | -0.26 |
| Acid abscisic (ABA; ng mg^-1^ DM) | 0.86 | 0.11 | -0.36 |
| Malate (area mg^-1^ DM) | 0.85 | 0.37 | -0.16 |
| Jasmonic Acid (JA; ng mg^-1^ DM) | 0.73 | -0.37 | -0.42 |
| Lactate (area mg^-1^ DM) | 0.73 | 0.56 | -0.19 |
| Trehalose (ng mg^-1^ DM) | 0.6 | 0.007 | 0.66 |
| Glucose (ng mg^-1^ DM) | 0.59 | 0.12 | 0.73 |
| Auxin (IAA; pg mg^-1^ DM) | -0.57 | -0.36 | -0.095 |
| Dihydroxybenzoic acid (DHBA; area mg^-1^ DM) | 0.57 | -0.57 | -0.19 |
| Sucrose (ng mg^-1^ DM) | 0.51 | -0.044 | 0.74 |
| Fumarate (area mg^-1^ DM) | 0.48 | 0.64 | -0.22 |
| Fructose (area mg^-1^ DM) | 0.46 | -0.11 | 0.81 |
| SA bound (ng mg^-1^ DM) | 0.39 | -0.71 | -0.013 |
| Succinate (area mg^-1^ DM) | 0.38 | 0.44 | -0.15 |
| Pyruvate (area mg^-1^ DM) | -0.33 | 0.43 | -0.11 |
| DHBA bound (area mg^-1^ DM) | 0.28 | -0.66 | 0.064 |
| Citrate (area mg^-1^ DM) | -0.23 | -0.015 | -0.27 |
| SA conjugates (area mg^-1^ DM) | 0.13 | -0.89 | -0.094 |
| DHBA xylose (area mg^-1^ DM) | 0.13 | -0.9 | -0.12 |
| Glutamine (ng mg^-1^ DM) | 0.09 | -0.0036 | -0.64 |
| Proline (ng mg^-1^ DM) | 0.09 | -0.02 | -0.73 |
