# Supplementary material for: The genetic interaction of REVOLUTA and WRKY53 links plant development, senescence, and immune responses
Source: PLoS One. 2022 Mar 25;17(3):e0254741. doi: 10.1371/journal.pone.0254741 (PMC8956159; doi:10.1371/journal.pone.0254741)
Supplement: S2 Table — Genetic interaction was analyzed by two-way anova performed on log10-transformed values of the different traits over the development. Metabolic compounds values were used at 15 days after flowering (F+15) and pathogen susceptibility was analyzed at 4 days after infection (DAI). The coefficient represents the effect of the presence of a single functional allele (REV or WRKY53), or both functional alleles REV*WRKY53, compared to mutated alleles. The p-value indicates the significance of the genetic interaction: ns = non significant,.: P ≤ 0.1, *: P ≤ 0.05, **: P ≤ 0.001, and ***: P ≤ 0.0001. B = bolting, F = flowering, F+10d = 10 days after flowering and S = mature silique stage; SA bound = total SA after hydrolysis—free SA, SA conjugates = SA-(C6)-glycosides, DHBA = dihydroxybenzoic acid; DHBA bound = Total DHBA after hydrolysis—free DHBA; cfu = colony forming units. (DOCX) [file pone.0254741.s002.docx]

**S2 Table. Coefficients of genetic interaction between *REV* and *WRKY53* on metabolic, developmental and immune traits.** Genetic interaction was analyzed by two-way anova performed on log_10_-transformed values of the different traits over the development. Metabolic compounds values were used at 15 days after flowering (F+15) and pathogen susceptibility was analyzed at 4 days after infection (DAI). The coefficient represents the effect of the presence of a single functional allele (*REV* or *WRKY53*), or both functional alleles *REV*WRKY53*, compared to mutated alleles. The *p*-value indicates the significance of the genetic interaction: ns = non significant, .: P ≤ 0.1, *: *P* ≤ 0.05, **: *P* ≤ 0.001, and ***:  *P* ≤ 0.0001. B = bolting, F = flowering, F+10d = 10 days after flowering and S = mature silique stage; SA bound = total SA after hydrolysis - free SA, SA conjugates = SA-(C6)-glycosides, DHBA = dihydroxybenzoic acid; DHBA bound = Total DHBA after hydrolysis - free DHBA; cfu = colony forming units.

|  | **Coefficient** | | | ***p-value*** | | | | | |
| --- | --- | --- | --- | --- | --- | --- | --- | --- | --- |
| **Trait** | ***REV*** | ***WRKY53*** | ***REV*WRKY53*** | ***REV*** | | ***WRKY53*** | | ***REV*WRKY53*** | |
| ***Developmental analysis*** |  |  |  |  |  |  |  |  |  |
| Rate of leaf production | 0,038 | -0,022 | 0,013 | 0,000 | *** | 0,001 | ** | 0,162 | ns |
| Leaf number (B) | 0,058 | -0,030 | 0,055 | 0,000 | *** | 0,016 | * | 0,002 | ** |
| Flowering time (F) | 0,001 | -0,007 | 0,029 | 0,900 | ns | 0,493 | ns | 0,038 | ** |
| Senescent area (F+10d) | -0,104 | -0,055 | 0,482 | 0,577 | ns | 0,768 | ns | 0,081 | . |
| Shoot dry mass (S) | -0,084 | -0,077 | 0,139 | 0,078 | . | 0,104 |  | 0,040 | * |
| Reproductive allocation (S) | 0,507 | 0,152 | -0,206 | 0,000 | *** | 0,082 | . | 0,091 | . |
| Number Silique (S) | 0,485 | 0,037 | -0,015 | 0,000 | *** | 0,485 | ns | 0,842 | ns |
|  |  |  |  |  |  |  |  |  |  |
| ***Metabolic analysis (F+15)*** |  |  |  |  |  |  |  |  |  |
| SA | 0,105 | -0,043 | 0,143 | 0,191 | ns | 0,581 | ns | 0,205 | ns |
| SA bound | 0,049 | -0,027 | 0,110 | 0,617 | ns | 0,783 | ns | 0,426 | ns |
| SA conjugates | 0,125 | 0,003 | 0,029 | 0,548 | ns | 0,988 | ns | 0,922 | ns |
| DHBA xylose | 0,076 | -0,015 | 0,065 | 0,687 | ns | 0,935 | ns | 0,808 | ns |
| DHBA bound | 0,158 | -0,051 | 0,116 | 0,114 | ns | 0,593 | ns | 0,398 | ns |
|  |  |  |  |  | ns |  |  |  |  |
| ***Pathogen assays (4 DAI)*** |  |  |  |  |  |  |  |  |  |
| log cfu cm-2 | -4,001 | -1,790 | 0,920 | 0,000 | ns | 0,008 | ** | 0,301 | ns |
