# Supplementary material for: The genetic interaction of REVOLUTA and WRKY53 links plant development, senescence, and immune responses
Source: PLoS One. 2022 Mar 25;17(3):e0254741. doi: 10.1371/journal.pone.0254741 (PMC8956159; doi:10.1371/journal.pone.0254741)
Supplement: S3 Table — Percentages indicate the percentage of the total variance explained in the three first principal components (PC). Loadings are correlation coefficients between the variables and PCs. (DOCX) [file pone.0254741.s003.docx]

**S3 Table. Loadings of the variables included in the PCA on mean of 14 growth-related traits values.** Percentages indicate the percentage of the total variance explained in the three first principal components (PC). Loadings are correlation coefficients between the variables and PCs.

| **Trait (unit)** | **PC1** | **PC2** | **PC3** |
| --- | --- | --- | --- |
|  | **40.2%** | **32.8%** | **9.5%** |
| Leaf number (leaf) | -0.94 | 0.05 | 0.02 |
| Leaf dry mass (g) | -0.94 | 0.08 | 0.25 |
| Bolting time (d) | -0.86 | -0.0039 | 0.14 |
| Flowering time (d) | -0.85 | -0.38 | 0.0062 |
| Specific leaf area (SLA; m^2^ g^-1^) | 0.78 | 0.46 | -0.23 |
| Whole-rosette area (cm^2^) | -0.76 | 0.5 | 0.14 |
| Rate of leaf production (RLP; leaf d^-1^) | -0.74 | 0.04 | -0.063 |
| Maximum rate of leaf expansion (R_max_; cm^2^ d^−1^) | 0.52 | 0.31 | 0.38 |
| Senescent area (%) | -0.27 | 0.62 | -0.68 |
| Stem dry mass (g) | -0.23 | 0.85 | 0.29 |
| Petiole dry mass (g) | -0.19 | -0.85 | -0.19 |
| Stem lenght (cm) | -0.11 | 0.91 | 0.17 |
| Reproductive allocation | 0.26 | 0.9 | 0.18 |
| Whole-rosette *F*_v_/*F*_m_ | 0.37 | -0.64 | 0.62 |
