# Supplementary material for: The genetic interaction of REVOLUTA and WRKY53 links plant development, senescence, and immune responses
Source: PLoS One. 2022 Mar 25;17(3):e0254741. doi: 10.1371/journal.pone.0254741 (PMC8956159; doi:10.1371/journal.pone.0254741)
Supplement: S4 Table — (DOCX) [file pone.0254741.s004.docx]

**S4 Table. List of primers used.**

| **Gene** | **Locus** | **Direction** | **Primer Sequence** |
| --- | --- | --- | --- |
| *ACTIN2* | At3g18780 | Forward | ACCCGATGGGCAAGTCATCACG |
|  |  | Reverse | TCCCACAAACGAGGGCTGGA |
| SAG13 | At2g29350 | Forward | AGGGAGCATCGTGCTCATATCC |
|  |  | Reverse | CCAGCTGATTCATGGCTCCTTTG |
| *S3H* | At4g10500 | Forward | AATATCGGCGACCAAATGCAGGTC |
|  |  | Reverse | ACTACGGCTCTATGGAGCACAC |
| *SID2* | At1g74710 | Forward | GCTTGGCTAGCACAGTTACAGC |
|  |  | Reverse | CACTGCAGACACCTAATTGAGTCC |
