# Supplementary figures and images for: The genetic interaction of REVOLUTA and WRKY53 links plant development, senescence, and immune responses
Source: PLoS One. 2022 Mar 25;17(3):e0254741. doi: 10.1371/journal.pone.0254741 (PMC8956159; doi:10.1371/journal.pone.0254741)

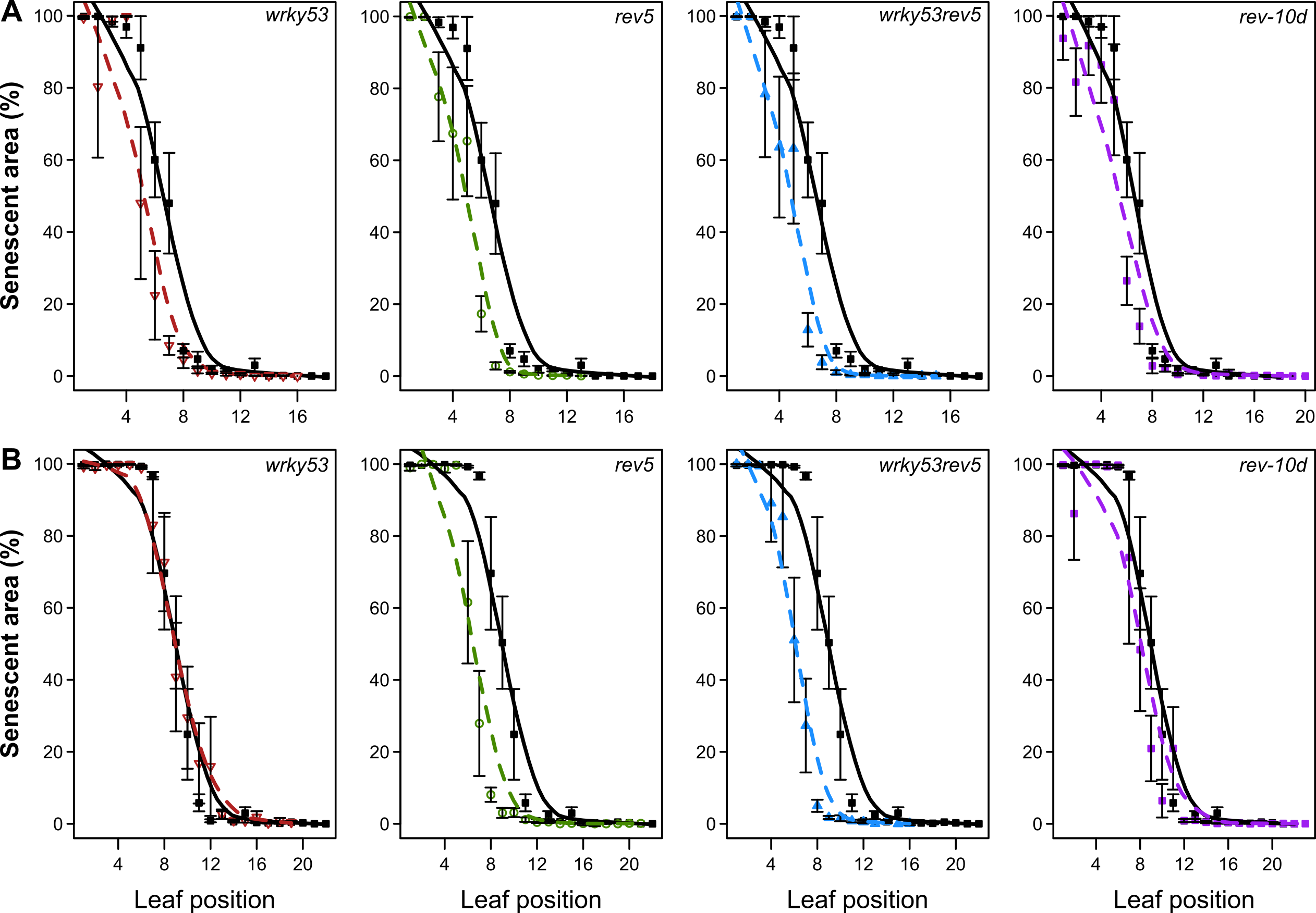

Supplement: S1 Fig — (A) Percentage of senescent area displayed at 10 days and (B) 15 days after flowering. Senescent area was calculated by the ratio between pixel number for Fv/Fm ≤ 0.6 and the total pixel number of the rosette. Black line means wild-type Col-0, colours show mutants. Data are means (± SE) of 5 plants. (TIF) [file pone.0254741.s005.tif]

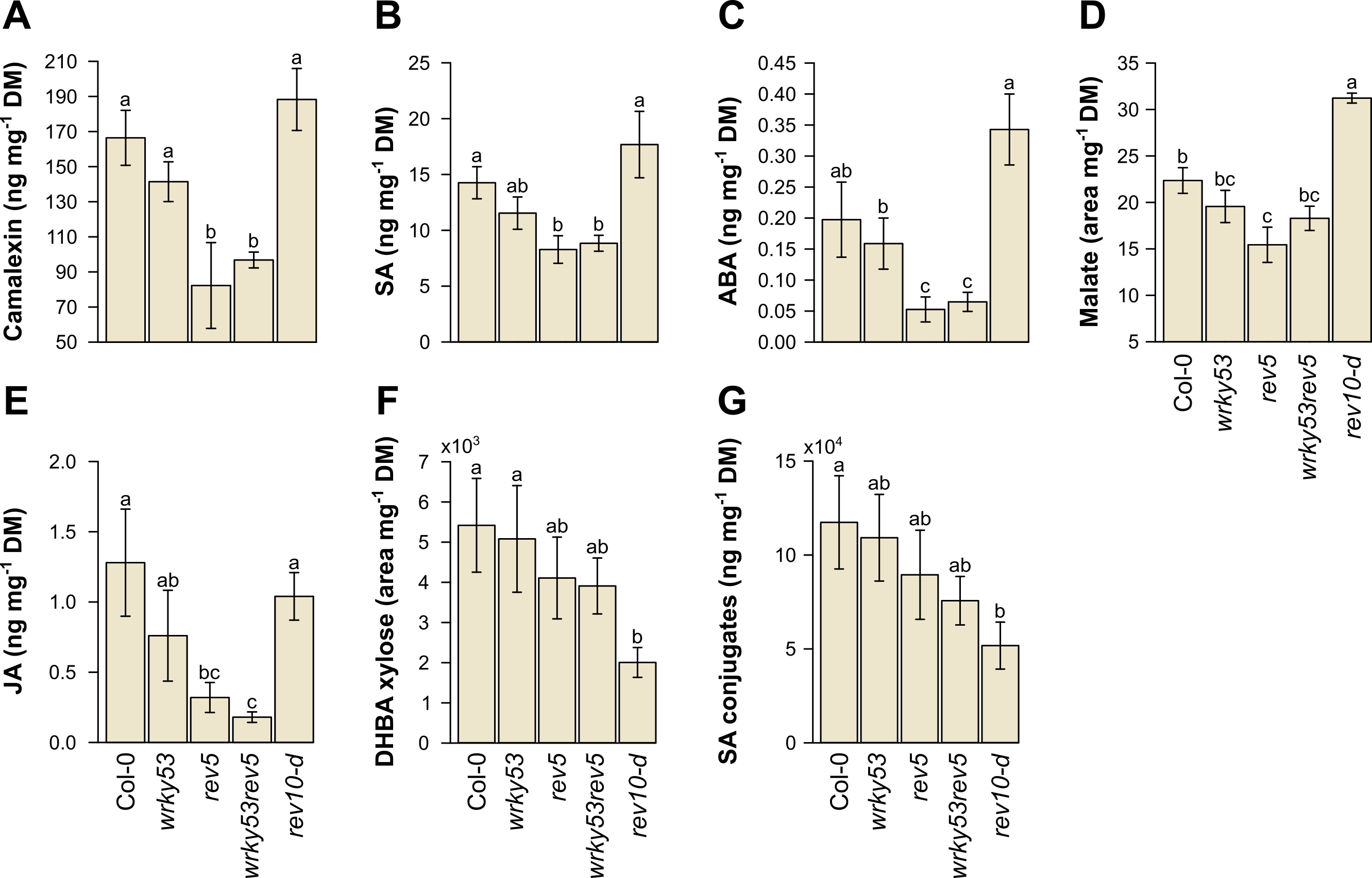

Supplement: S2 Fig — (A) camalexin, (B) salicylic acid (SA), (C) abscisic acid (ABA), (D) malate, (E) jasmonic acid (JA), (F) dihydroxybenzoic acid (DHBA) xylose and (G) SA conjugates. Data are means (±SE) of 5 plants. Different letters indicate significant differences between means following Kruskal-Wallis tests (P < 0.05). DM = dry mass; SA conjugates = SA-(C6)-glycosides. (TIF) [file pone.0254741.s006.tif]

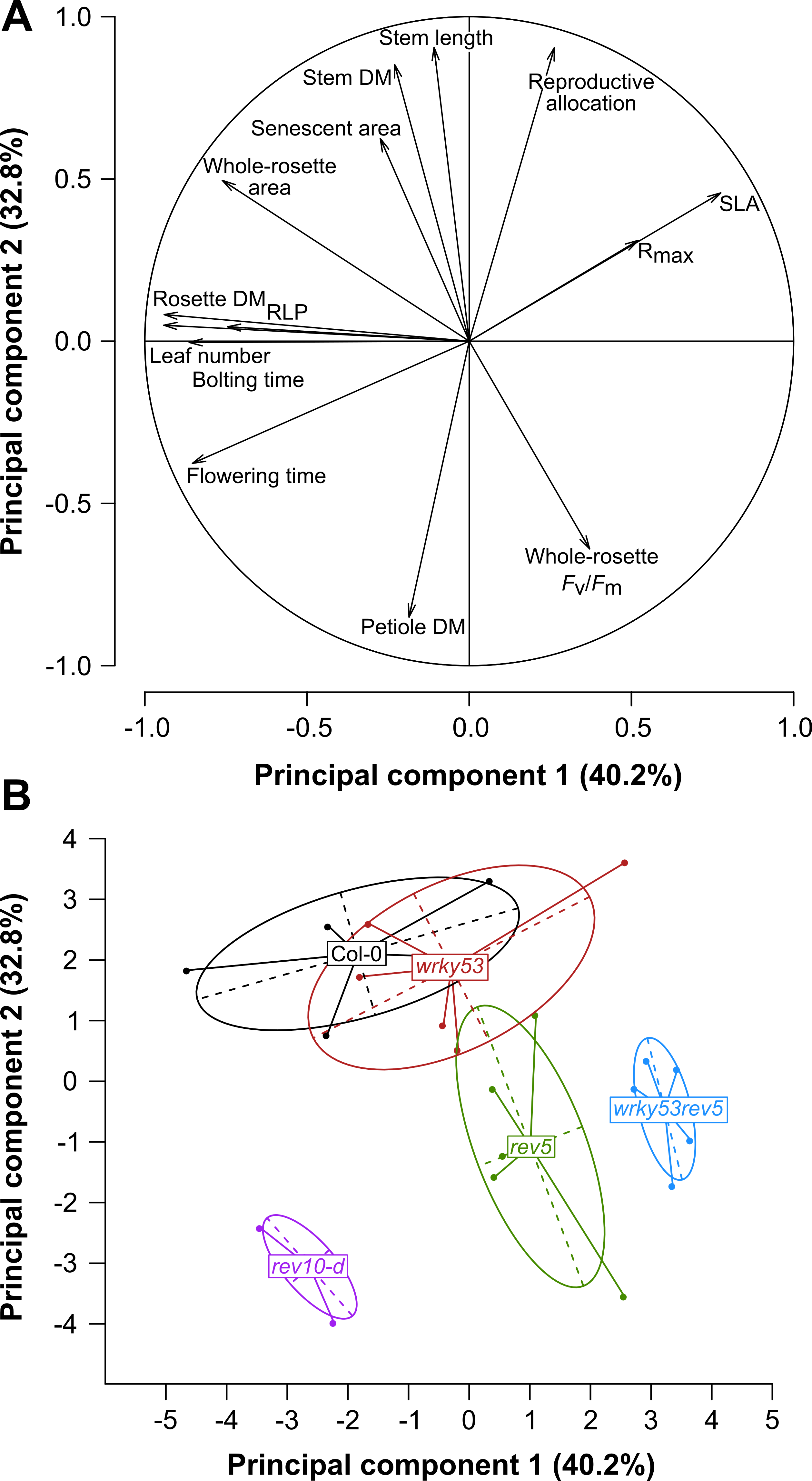

Supplement: S3 Fig — (A) Representation of the variables, measured at 10 days after flowering, on the two first principal components. DM = dry mass; RLP = rate of leaf production; Rmax = maximum rate of leaf expansion; SLA = specific leaf area; Fv/Fm = maximum quantum yield of photosystem II. (B) Projection of individual plants with centres of gravity per genotype (n = 5). Ellipses represent inertia ellipses, centred on the means for each genotype. Their width and height are given by 1.5 times the standard deviation of the coordinates on axes, and the covariance sets the slope of the main axis [58]. rev5 and wrky53 are single knock-out of the REV and WRKY53 genes, respectively. wrky53rev5 is double knock-out (Xie et al., 2014). rev10-d a semi-dominant gain-of-function of REV allele where REV mRNA is rendered resistant to the negative regulation by microRNAs [27]. (TIF) [file pone.0254741.s007.tif]

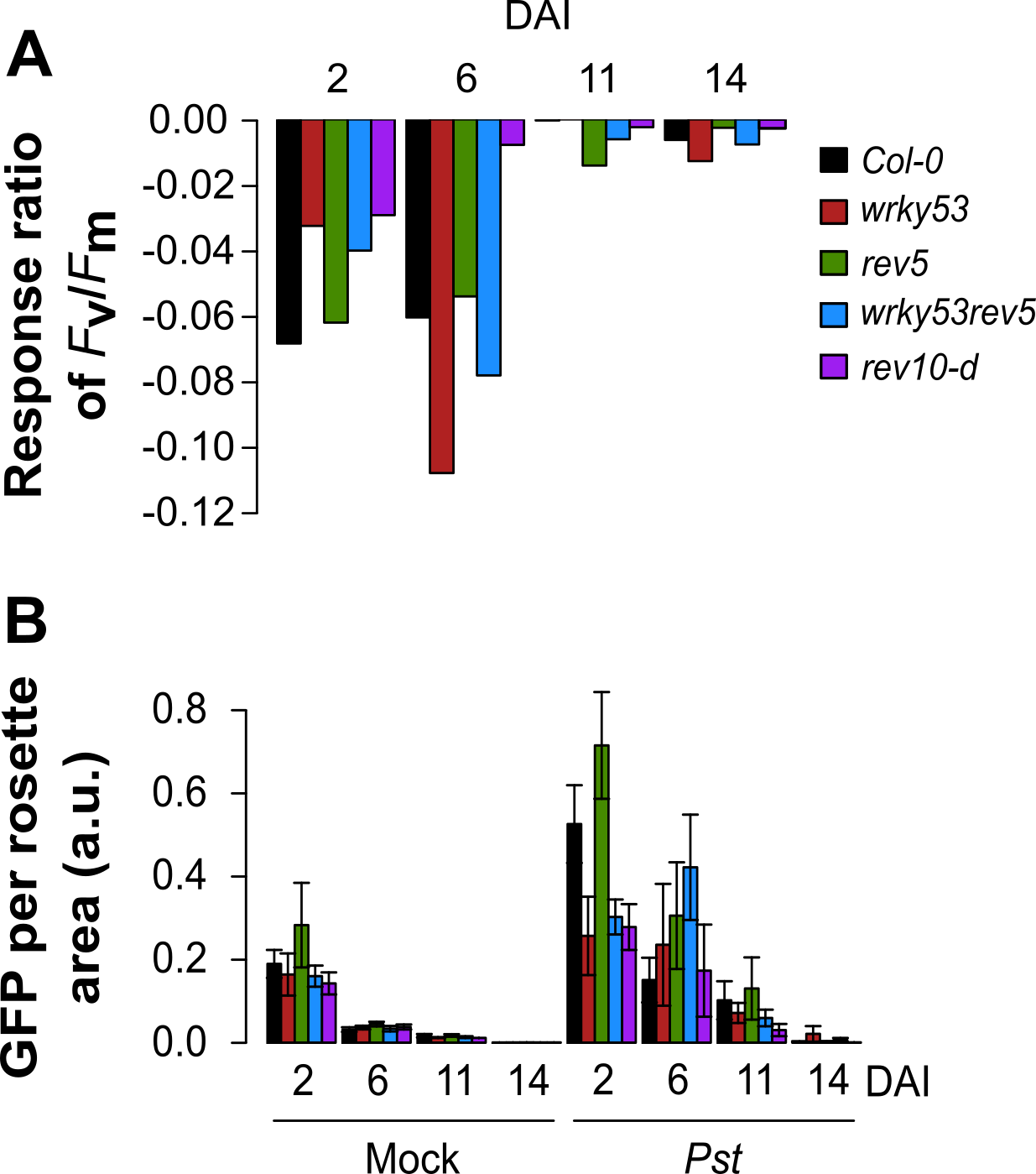

Supplement: S4 Fig — 16-day-old plants were infected with Pseudomonas syringae (Pst) pv tomato DC3000, constitutively expressing GFP by spraying a Pst suspension on leaf surfaces. (A) Response ratio of photosynthetic efficiency (Fv/Fm) during days after infection (DAI), calculated as the relative ratio of Pst-infected plants compared to mock-treated plants (Pst-Mock)/Mock. (B) Quantification of bacterial growth in planta expressed in GFP units per rosette area compared to the respective mock condition. Data are means (± SE) of 3–5 plants per condition. (TIF) [file pone.0254741.s008.tif]
