# Supplementary material for: The genetic interaction of REVOLUTA and WRKY53 links plant development, senescence, and immune responses
Source: PLoS One. 2022 Mar 25;17(3):e0254741. doi: 10.1371/journal.pone.0254741 (PMC8956159; doi:10.1371/journal.pone.0254741)
Supplement: S1 File — (DOCX) [file pone.0254741.s009.docx]

**S1 File**

**Targeted metabolomic analysis**

The targeted LC-MS profiling analysis was performed using an Ekspert Micro-LC 200 and a QTRAP4000 (ABSciex). Chromatographic separation was achieved on a Halo C18 column (150 x 0.5 mm; 2.7 µm; 90A; Eksigent) with a column temperature of 35 °C applying the following binary gradient at a flow rate of 11 µL/min: 0 - 0.1 min isocratic 95 % A; 0.1 - 3 min, linear from 95 % A to 5 % A; 3 - 3.8 min, isocratic 5 % A (water, 0.1 % FA); 3.8 - 4 min, linear from 5 % A to 95 % A; 4 - 5 min, isocratic 5 % B (acetonitrile, 0.1 % FA). The injection volume was 2 µL. Analytes were ionized using a TurboV ion source equipped with an Assy 65 µm ESI electrode in positive (ionspray voltage 5000 V) and negative ion mode (ionspray voltage -4500 V). The following instrument settings were applied: nebulizer and heater gas, nitrogen, 25 and 10 psi; curtain gas, nitrogen, 20 psi; collision gas, nitrogen, medium; source temperature, 200°C; entrance potential, 10 V; collision cell exit potential, 5V; scan time 200 msec. The transitions monitored for each analyte were indicated in the following table.

**Table below: Parameters of targeted metabolomic measurements.**First mass (Q1) and second mass (Q3) analyzers of triple quadrupole mass spectrometers. The declustering potential is a voltage applied to the orifice that helps to prevent the ions from clustering together. The c**ollision energy** is the potential difference between Q0 and Q2 (collision cell).

| **Q1 mass (Da)** | **Q3 mass (Da)** | **Declustering potential (volts)** | **Collision energy**  **(volts)** | **Compound** | **Quantifier ion** |
| --- | --- | --- | --- | --- | --- |
| 137 | 93 | -36 | -10 | Salicylic acid (SA 1) | - |
| 137 | 65 | -36 | -40 | Salicylic acid (SA 2) | + |
| 263 | 153 | -20 | -16 | Abscisic acid (ABA 1) | + |
| 263 | 219 | -20 | -16 | Abscisic acid (ABA 2) | - |
| 209.1 | 59 | -30 | -30 | Jasmonic acid (JA) | + |
| 299 | 137 | -50 | -15 | SA conjugate (+ hexose) | + |
| 153 | 109.03 | -36 | -17 | Dihydroxy bencoic acid (DHBA) | + |
| 315.1 | 153 | -50 | -15 | DHBA conjugate (+ hexose) | + |
| 285.1 | 153 | -50 | -10 | DHBA conjugate (+ xylose) | + |
| 201 | 142 | 60 | 20 | Camalexin (CAM 1) | - |
| 201 | 59 | 60 | 30 | Camalexin (CAM 2) | + |
| 176 | 130 | 30 | 18 | Indole acetic acid (IAA 1) | + |
| 176 | 148 | 30 | 20 | Indole acetic acid (IAA 2) | - |

**Untargeted metabolomic profiling LC-MS analysis**

For untargeted LC-MS analysis a Synapt G2 mass spectrometer equipped with an Acquity UPLC (Waters, Milford, MA, USA) was used. Chromatography was conducted on a Waters Acquitiy C_18_ HSS T3 column (100 x 2.1 mm, 1.8 µm) with a 10 min gradient from 98 % water with 0.1 % FA to 100 % MeOH with 0.1 % FA at 30 °C. In addition to the reversed phase chromatography a Cogent Diamond Hydride column (100 x 2.1 mm, 4 µm; MicroSolv Technology) was used for the separation of more polar compounds. Chromatography was performed with a flow rate of 400 µL/ min at 50 °C. A 20 min gradient from 0 - 13 min linear from 100 % B (acetonitrile; 0.1 % NH4 acetate; 0.1 % acetic acid v/v) to 40 % B, 13 - 14 min linear from 40 % B to 20 % B, 14 - 14.8 min linear to 0 % A (water; 0.1 % NH4 acetate; 0.1 % acetic acid v/v) and 14.8 - 20 min isocratic 100 % B. For both columns positive and negative electrospray ionisation modes were used at 3000 V ion spray voltage in positive and 2500 V in negative mode. The mass spectrometer was operated in MS and MS^E^ Resolution mode with a scan time of 0.5 sec and a scan range from m/z 50 - 2000. Data analysis was carried out using Waters MarkerLynx, EZinfo and Target Lynx software packages.

**GC-MS analysis**

The dried extracts were derivatised with 40 µL methoxamine in pyridine (20 mg/mL) for 90 min at 30 °C followed by an addition of 60 µL MSTFA and an additional incubation for 60 min at 40 °C. 1 µL of the resulting 100 µL total volume was submitted to GC-MS analysis (Agilent 6890 GC coupled to an Agilent 5973 single quad mass spectrometer). The GC-MS instrument was operated in splitless and splitted injection mode depending on the compound concentrations expected. Separation was achieved by a HP-5MS column (30 m, I.D. 0.25 mm, film 0.25 µm). The GC oven temperature was held at 70 °C for 5 min, then ramped at 15 °C/min to 300 °C, afterwards ramped at 75 °C/min to 310 °C and then held for an additional 10 min at 310 °C. Helium was used as carrier gas with a flow rate of 1 mL/min. Detection of analytes was performed by electron ionization (EI) single quadrupole mass spectrometry operated in selected ion monitoring (SIM) mode (see information of selected ions below).

**Table below: Parameters of GC-MS measurements**Two derivative type were used: trimethylsilyl (TMS) and methoximation reaction (MeOx) derivatisation. Retention time is the time elapsed between sample introduction (beginning of the chromatogram) and the maximum signal of the given compound at the detector.

| **Ions** | **Derivative Type** | **MM** | **Quantitative** | **Qualitative** | **Retention time (min)** |
| --- | --- | --- | --- | --- | --- |
| L-Prolin | 2TMS | 259 | 142 | 216 | 11.017 |
| Salicylic acid | 2TMS | 282 | 267.1 | 193.1 | 13.027 |
| 3 Hydroxybenzoic acid | 2TMS | 282 | 267.1 | 282.2 | 13.447 |
| Glutamic acid | 3TMS | 363 | 246 | 348 | 13.886 |
| Fructose | 5TMS MeOx |  | 307 | 217 | 15.882 and 15.949 |
| Glucose | 5TMS MeOx |  | 205 | 319 | 16.069 and 16.203 |
| Sucrose | 8TMS | 918 | 361.3 | 437.3 | 20.472 |
| Trehalose | 8TMS | 918 | 191.1 | 271.2 | 20.976 |
